# Resistant starches from dietary pulses modulate the gut metabolome in association with microbiome in a humanized murine model of ageing

**DOI:** 10.1038/s41598-023-37036-w

**Published:** 2023-06-29

**Authors:** Saurabh Kadyan, Gwoncheol Park, Bo Wang, Prashant Singh, Bahram Arjmandi, Ravinder Nagpal

**Affiliations:** 1 Department of Nutrition and Integrative Physiology, College of Health and Human Sciences, Florida State University, Tallahassee, FL 32306 USA; 2 Department of Biomedical and Chemical Engineering and Sciences, Florida Institute of Technology, Melbourne, FL 32901 USA

**Keywords:** Metabolomics, Nutrition, Metabolic disorders, Next-generation sequencing, Microbiome

## Abstract

Emerging evidence suggests that plant-based fiber-rich diets improve ageing-associated health by fostering a healthier gut microbiome and microbial metabolites. However, such effects and mechanisms of resistant starches from dietary pulses remain underexplored. Herein, we examine the prebiotic effects of dietary pulses-derived resistant starch (RS) on gut metabolome in older (60-week old) mice carrying a human microbiome. Gut metabolome and its association with microbiome are examined after 20-weeks feeding of a western-style diet (control; CTL) fortified (5% w/w) with RS from pinto beans (PTB), black-eyed-peas (BEP), lentils (LEN), chickpeas (CKP), or inulin (INU; reference control). NMR spectroscopy-based untargeted metabolomic analysis yield differential abundance linking phenotypic differences in specific metabolites among different RS groups. LEN and CKP increase butyrate, while INU promotes propionate. Conversely, bile acids and cholesterol are reduced in prebiotic groups along with suppressed choline-to-trimethylamine conversion by LEN and CKP, whereas amino acid metabolism is positively altered. Multi-omics microbiome-metabolome interactions reveal an association of beneficial metabolites with the Lactobacilli group, *Bacteroides*, *Dubosiella*, *Parasutterella,* and *Parabacteroide*s, while harmful metabolites correlate with *Butyricimonas, Faecalibaculum, Colidextribacter, Enterococcus, Akkermansia, Odoribacter,* and *Bilophila*. These findings demonstrate the functional effects of pulses-derived RS on gut microbial metabolism and their beneficial physiologic responses in an aged host.

## Introduction

The proportion of world’s population aged 60 years and above is expected to double by the year 2050^1^. This underscores the need for preventive strategies aimed at reducing risk of chronic cardiometabolic and neurocognitive disorders among the elderly due to their senesced immunity and increased vulnerability to nutritional risk. Promoting healthspan in elderly will not only reduce their susceptibility to diseases but also curtail the rising cost of medical treatments^2^. Emerging evidence highlights the fundamental role of the gut microbiome in host immune and metabolic health, conferring resilience to various intestinal and cardiometabolic diseases throughout the lifespan^3,4^. Gut dysbiosis, characterized by decreased abundance of beneficial microbes such as short-chain fatty acids (SCFAs)-producing bacteria, overgrowth of pathobionts, and accumulation of detrimental metabolites in the gut environment, leads to impaired resilience against non-communicable diseases (NCDs)^5^. Although there are several hallmarks of ageing process, research in past decade focusing on biology of ageing has proposed microbiome disturbance as one of the crucial contributory factors behind ageing-related health loss^6^. The ageing process is associated with the age-dependent depletion of beneficial genera (e.g., *Bifidobacteria*) and an increased population of opportunistic/pathogenic bacteria (e.g., members of phylum Proteobacteria) in the human gut^7^. Diet is one of the strongest regulators and modulators of gut microbiome. While a western-style diet can induce gut dysbiosis, a fiber-rich diet can reverse at least partly, these impairments^8^. Thus, regulating and fostering a healthier gut microbiome through a prudent dietary regimen could be a proactive strategy for improving the overall health of the elderly.

Dietary fibers exert physiological responses by acting as prebiotics. They remain largely undigested in the upper intestine and are fermented by colonic microbiota, leading to the generation of beneficial metabolites that positively influence host health^9^. Incorporating ‘nutrient-dense’ dietary pulses in the daily diet can enrich the dietary fiber content, conferring specific health benefits to the host^10–12^. Importantly, dietary pulses-derived starch, previously considered a major by-product of commercial protein extraction from pulses, has recently received remarkable interest owing to its prebiotic potential after its conversion to resistant starch (RS)^2,13^. RS offers precision modulation of the gut microbiota-metabolite interplay, which may depend upon their discrete structures and types: RS type-1 (physically-inaccessible starch), RS type-2 (native-ungelatinized starch), RS type-3 (retrograded starch), RS type-4 (chemically-modified starch), and RS type-5 (starch-lipid complexes)^14^. RS exerts various physiological effects via its microbial fermentation to SCFAs (butyrate, acetate, and propionate) in the colon. Acetate, a major SCFA, inhibits pathobionts’ growth by reducing colonic pH; butyrate, on the other hand, possesses immune-modulatory properties and plays a central role in furnishing energy to colonocytes, thus maintaining intestinal integrity; lastly, propionate acts as a gluconeogenic substrate after being translocated to the liver, improving glucose metabolism and aiding in lowering blood cholesterol levels^2,15^.

To gain deeper insights into the functional fingerprints of RS on host-microbiota interactions, an integrated multi-omics approach utilizing metabolomics and metagenomics is crucial to uncover key microbiota-associated metabolites that may mediate distinct beneficial effects to the host^16^. While studies have linked gut microbiome-metabolome modulations with health benefits associated with cereals- and tubers-derived RS, such effects pertaining to pulses-derived RS have been mainly reported in in-vitro fermentation models, and in-vivo mechanisms remain unclear^17–20^. Moreover, to the best of our knowledge, no data exist on the alteration in microbial metabolic processes upon consumption of pulses-derived RS in aged hosts, who are otherwise at greater risk of nutritional deficiencies. In our recent studies, we demonstrated that these RSs ameliorate gut and metabolic health by enhancing intestinal epithelial integrity, reducing inflammation, and fostering beneficial and SCFAs-producing microbiome clades in a humanized murine model of aging^21^. These findings propelled us to further investigate the functional lineaments of the gut microbiome to gain deeper insights into their prebiotic effects and mechanisms. In this addendum study, we aim to examine how the incorporation of RS from different dietary pulses in a western-style diet modulates the gut metabolome in ageing mice carrying the human microbiota. We also integrate and examine metabolomic-microbiome interplay to generate diverse metabolite footprints. Our results reveal novel and distinct signatures of gut microbial metabolites associated with SCFAs production, altered bile acid and amino acid metabolism, and specific mutualistic and competitive interactions across different taxa. These finding highlight the potential of discrete structures of these dietary fibers to induce targeted alterations in gut metabolomic pool and advance our understanding of the function and performance of the gut microbiome using RS intervention, particularly in the ageing gut.

## Results

### Resistant starches derived from different dietary pulses distinctly modulate the gut metabolomic arrays

**Figure 1:**
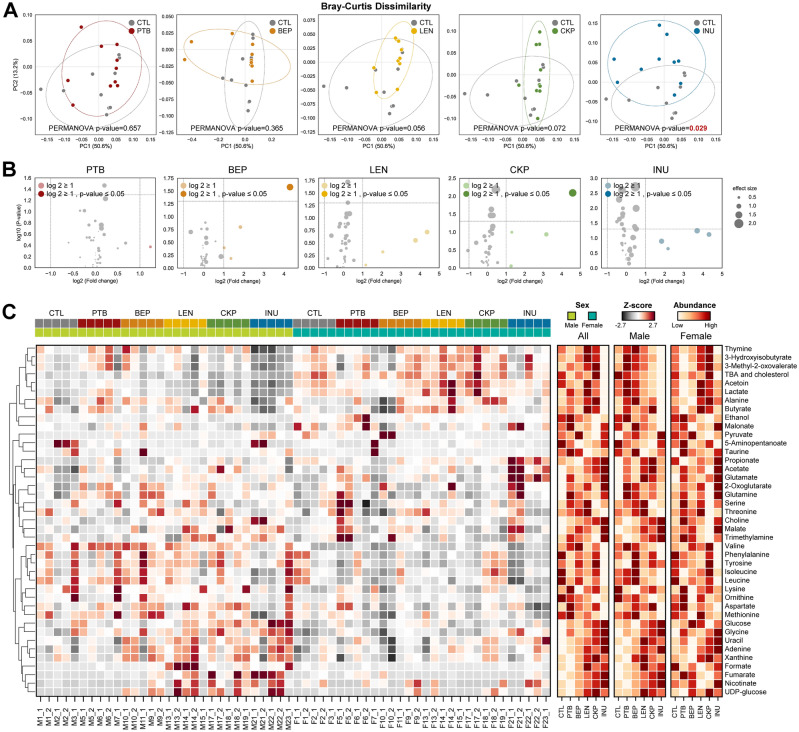
Resistant starches derived from different dietary pulses distinctly modulate the gut metabolomic arrays. Prebiotic effects of dietary fiber (resistant starches versus inulin) in modulating the gut metabolomics profiles in older mice colonized with human gut microbiome. (**A**) PCoA analysis (Bray–Curtis dissimilarity index); (**B**) Volcano plot showing differential metabolites (fold-change ≥ 1; *P* ≤ 0.05 in dark color; and FC ≥ 1; *P* > 0.05 in light color); (**C**) The abundance profiles transformed to Z scores and rank of the groups in all, male, and female mice. *CTL* control western-style diet group, *PTB* pinto beans, *BEP* black-eyed peas, *LEN* lentils, *INU* inulin.

Principal coordinate analysis (PCoA) of the NMR-based fecal metabolomics data reveals specific variation patterns in the metabolite profiles of treatment groups compared to CTL (Fig. 1A). Although no significant differences in the clustering of PTB and BEP are observed, LEN (*p* = 0.056) and CKP (*p* = 0.072) explain considerable variation in the metabolomics arrays relative to CTL. In comparison, INU generates a significantly distinct (*p* = 0.029) metabolite profile compared to CTL. Subsequently, we apply Log2-fold change (FC) analysis on differential metabolites, ascertained using Volcano plots (Fig. 1B). Amongst all groups, only one metabolite in each of BEP and CKP exhibits significant FC ≥ 1 (*p* < 0.05) compared to CTL. Additionally, the metabolites with FC ≥ 1 but insignificant p-value are arranged in the following ascending order: INU = LEN (4) > CKP = BEP (3) > PTB (1). We analyze the metabolites’ abundance in individual samples using Z-scores and depict them in a heatmap, wherein each molecule is ranked based on abundance for combined and separate sexes (Fig. 1C). Broadly, metabolites including acetoin, lactate, total bile acids (TBAs), and cholesterol yield distinct clusters of abundance in females, while valine, phenylalanine, tyrosine, isoleucine, and leucine predominate males. Overall rank scores yield distinct arrays in the number and dynamics of abundant metabolites after dietary intervention.

### Specific gut metabolomic signatures associate with resistant starches from different dietary pulses

**Figure 2:**
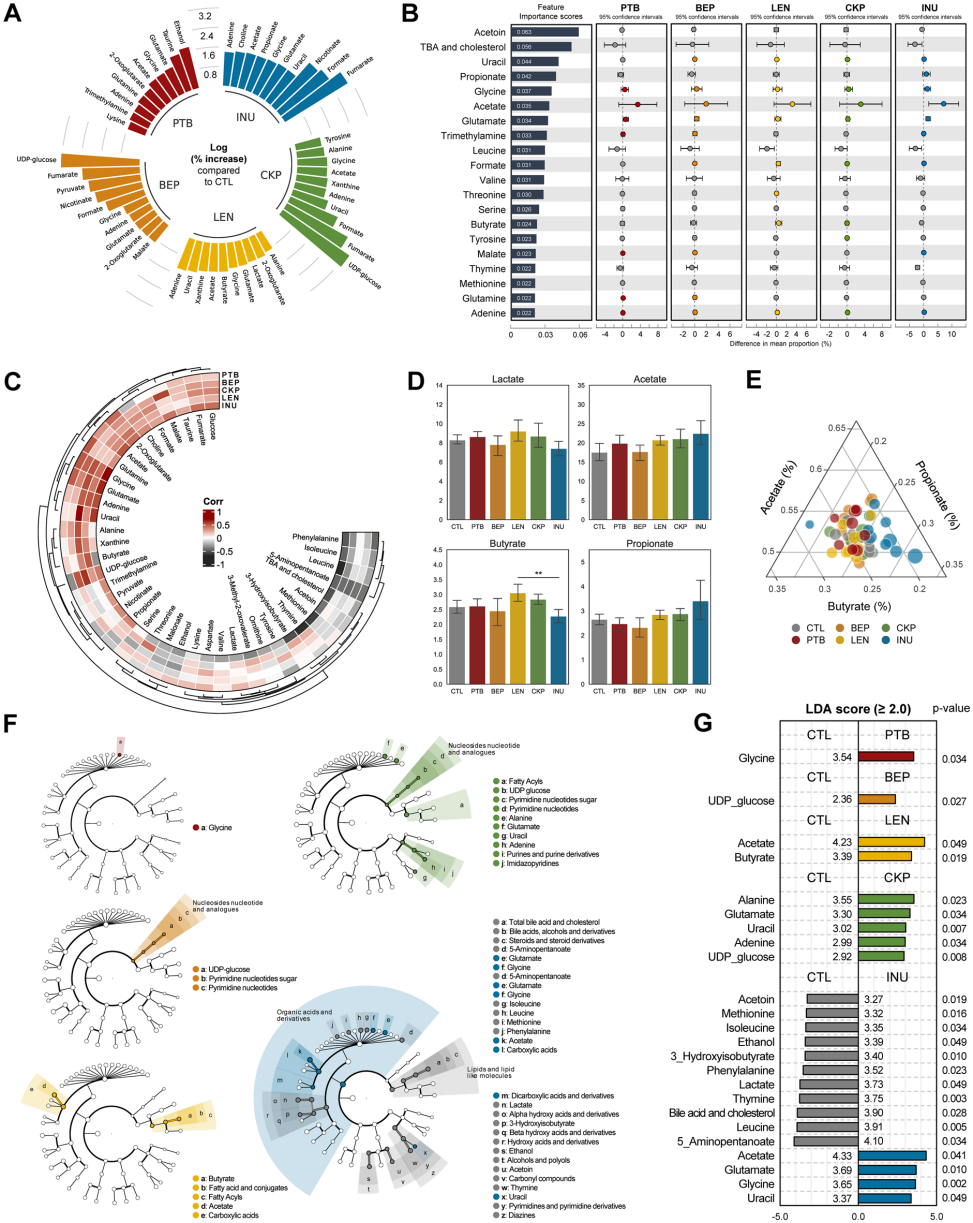
Specific gut metabolomic signatures associate with resistant starches from different dietary pulses. Prediction, correlation, and identification of gut metabolites specific to dietary fiber (resistant starch or inulin) relative to standard western-style diet group. (**A**) Metabolites with the greatest increase (% log change) in each group. (**B**) Top 20 most strongly predictive metabolites based on relative importance score used to assess the contribution to classifier accuracy, and extended error bar plots for those taxa between control, resistant starch, and inulin groups. Corrected *p* value (Welch’s two-sided *t* test) are shown in here. (**C**) Correlation of metabolites in resistant starches versus inulin groups relative to control group. (**D**) Relative abundance of lactates and SCFAs (acetate, butyrate, propionate). (**E**) Ternary plot showing the ratio of the three major SCFAs. Ratio is calculated after log-transformation of values; the marker size is determined by the sum of three SCFAs. (**F**) LEfSe cladogram: the four levels of hierarchy are based on the chemical taxonomy of Human Metabolome Database (HMDB). (**G**) LEfSe scores (LDA ≥ 2; *P* < 0.05). *CTL* control western-style diet group, *PTB* pinto beans, *BEP* black-eyed peas, *LEN* lentils, *INU* inulin.

Firstly, we shortlisted and identified the top 10 metabolites that exhibited the greatest increase (% log change) in individual RS groups compared to the CTL group, as presented in Fig. 2A. We observed several group-specific metabolites such as ethanol and taurine [PTB]; UDP-glucose [BEP, CKP], fumarate [BEP, CKP, INU], and nicotinate [BEP, INU], which exhibit a high increase. Subsequent analysis in terms of feature importance scores to observe top 20 strongly predictive and discriminatory metabolites among different RS groups versus CTL also yield distinct arrays of metabolites (Fig. 2B), some of which are unique from the % log change arrays. Specifically, glycine, acetate, glutamate, and adenine shared prediction for all treatment groups, whereas acetoin, leucine, serine, thymine, methionine, TBAs, and cholesterol predict specifically for the CTL group. Among SCFAs, propionate predicts only for INU while butyrate is involved with LEN and CKP groups.

Subsequent correlation analysis reveals the association of specific metabolites with different treatment groups (Fig. 2C), aligning well with earlier rank assessment and feature important scores. Metabolites including glucose, fumarate, 2-oxoglutarate, acetate, glutamine, glycine, glutamate, adenine, and uracil correlate positively with RS groups, while phenylalanine, isoleucine, leucine, 5-aminopentanoate, acetoin, methionine, TBAs, and cholesterol show negative correlation. UDP-glucose and choline show a direct correlation with all RS groups except PTB. Notably, butyrate exhibits strong positive correlation with LEN and CKP, but negative association with INU. Propionate shows a strong positive correlation with INU, weaker positive association with LEN and CKP, and negative association with PTB and BEP. The changes in the proportion (normalized) of SCFAs for each group are further visualized in Fig. 2D, wherein lactate and butyrate are highest in LEN followed by CKP while acetate and propionate are highest in INU. Besides, butyrate in LEN is significantly abundant (*p* < 0.01) compared to INU. A ternary plot further highlights the proportion of these SCFAs varying among RS versus CTL groups (Fig. 2E), with INU samples forming divergent clusters of low butyrate and high propionate levels.

Moving further, the execution of biomarkers discovery algorithm i.e., the linear discriminant analysis effect size (LefSe)-based cladogram, demonstrates distinct hierarchical clusters of chemical taxonomy (devised as per human metabolome database) that are upregulated or downregulated in RSs versus CTL groups (Fig. 2F). The LefSe analysis identifies several significant (LDA score ≥ 2.0; *p* < 0.05) discriminant metabolites associated with each group (Fig. 2G). Glycine and UDP-glucose are the only metabolites upregulated in PTB and BEP, respectively. LEN significantly enhances acetate and butyrate each belonging to clades of carboxylic acids and fatty acyls, respectively. CKP demonstrates an abundance of amino acids (alanine, glutamate) and nucleic acid derivatives (UDP-glucose and adenine). Interestingly, CKP exhibits no significant abundance of individual SCFAs; however, the cladogram reveals an overall enrichment of fatty acyls clade. INU significantly alters the metabolomic pool relative to CTL, with overall enhancement of metabolites belonging to organic acids and derivatives (acetate, glycine, glutamate) and reduction in metabolites belonging to lipid and lipid-like molecules (TBAs and cholesterol), alcohols (ethanol) and carbonyl compounds (acetoin).

### Integrated multi-omics analyses reveal RS-specific modulations in microbiome-metabolome correlation networks

**Figure 3:**
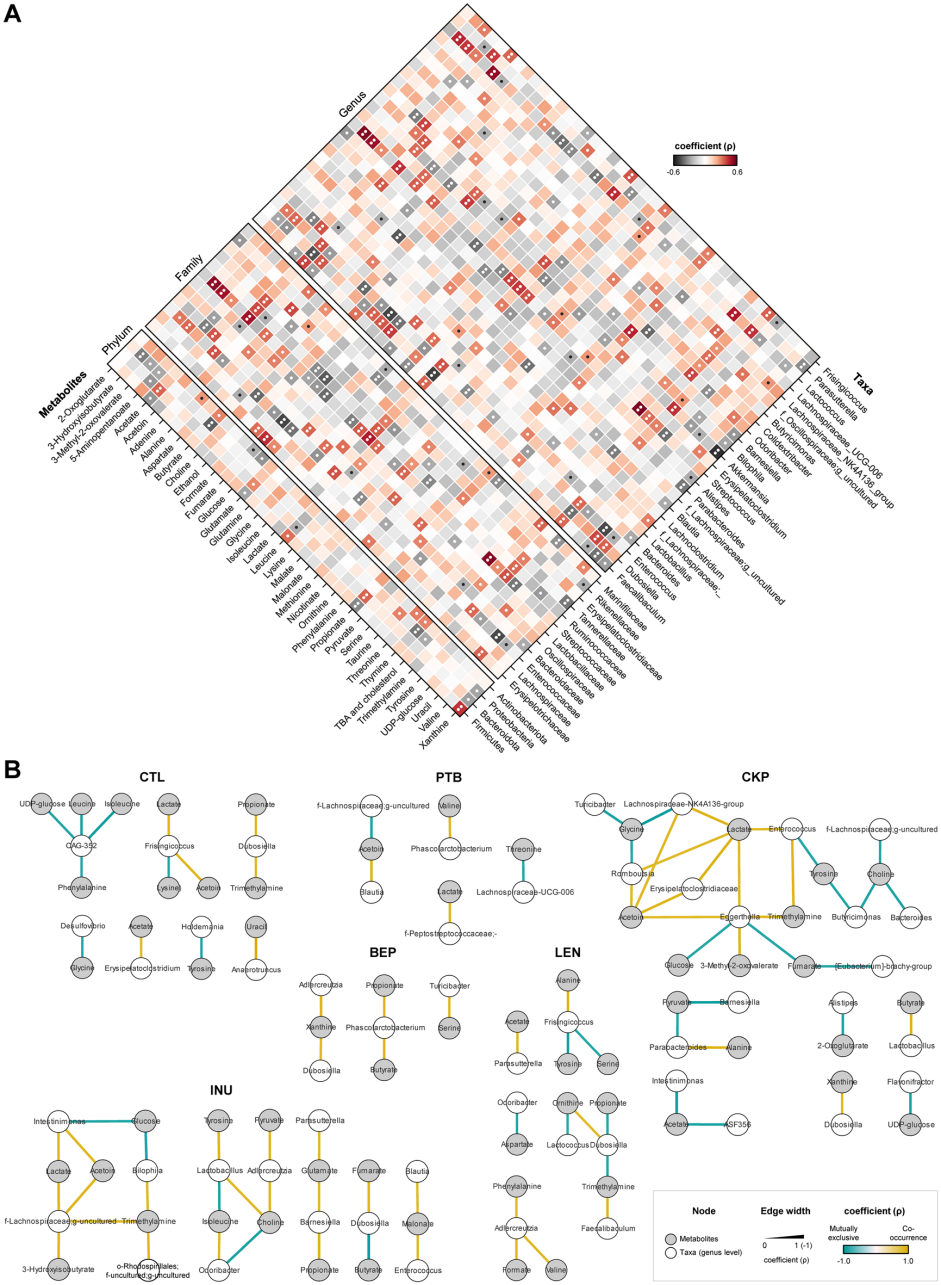
Integrated multi-omics analyses reveal modulations in microbiome-metabolome correlation networks specific to resistant starches from different dietary pulses. Microbiome-metabolome correlation of dietary fiber (resistant starch or inulin) relative to standard western-style diet group. (**A**) Correlation of gut metabolites with the major bacterial phyla, families and genera (*P < 0.05; **P < 0.01). (**B**) Microbiome-metabolome correlation network based on Spearman’s rank correlation coefficients. Each node represents one genus (white) or metabolite (gray); two nodes are linked if the correlation coefficient value is > 0.85 and P (FDR-corrected) is < 0.01. CTL: control western-style diet group; *PTB* pinto beans, *BEP* black-eyed peas, *LEN* lentils, *INU* inulin.

Recently, we demonstrated how RS differently modulate the gut microbiome in mice^21^. To explain the functional effects of microbiome modulation on metabolomic fingerprints, we herein integrated the two datasets and applied correlational analysis between major RS-modulated taxa (4 phyla, 12 families and 25 genera) and 41 microbial metabolites (Fig. 3A) to understand RS-specific modulation of the microbiome-metabolome correlation networks (Fig. 3B).

At the phylum level, Firmicutes is significantly correlated with a higher abundance of xanthine and leucine and a reduced abundance of glutamate, propionate, and acetate, whereas the enrichment trend for these metabolites is inverse for Bacteroidota. Proteobacteria exhibit a strong negative correlation with butyrate, 3-hydroxyisobutyrate, thymine, 3-methyl-2-oxovalerate, 5-aminopentanoate, xanthine, TBAs, and cholesterol. Actinobacteria demonstrate a positive association with alanine, threonine, and thymine, while negatively influencing fumarate and glucose. At the family level, *Streptococcaceae* impacts 18 metabolites, exhibiting a strong positive correlation (*p* < 0.01) with metabolites including trimethylamine (TMA), thymine, isoleucine, leucine, lactate, alanine and acetoin, and a negative correlation with glucose, glycine, and tyrosine. *Enterococcaceae* influences 17 metabolites, exhibiting a strong negative correlation with uracil, glucose, fumarate and choline, and a positive correlation with lactate, acetoin, TMA, and 3-methyl-2-oxovalerate. Family taxa directly associated with an increasing TBAs and cholesterol include *Oscillospiraceae*, *Streptococcaceae,* and *Marinifilaceae*. Propionate is positively associated with *Bacteroidaceae* and *Tannerellaceae. Ruminococcaceae* shows a strong positive correlation with TMA, lactate, acetoin, and a negative correlation with formate, fumarate and nicotinate. *Bacteroidaceae* show a positive association with acetate, propionate, glutamate, and glycine, whereas 5-aminopentanoate, leucine, thymine, and xanthine are negatively associated. *Lactobacillaceae* correlates with a decreased abundance of serine, ethanol, and acetoin but with an increased abundance of butyrate.

Amongst genera, the metabolomics pool is prominently influenced by the *Streptococcus* (18 metabolites) and *Lactococcus* (18), followed by *Enterococcus* (17), *Dubosiella* (14), *Akkermansia* (12), *Faecalibaculum* (11), *Bilophila* (11*), Bacteroides* (8), *Blautia* (8), *Frisingicoccus* (8), and *Parabacteriodes* (8). Acetoin (10 taxa), xanthine (10), TMA (9), lactate (8), propionate (8) leucine (7), and valine (7) are the most influenced metabolites across all taxa. TBAs and cholesterol, which strongly associate with CTL, are positively associated with *Butyricomonas, Colidextribacter, Odoribacter,* and *Streptococcus,* but negatively correlated with *Blautia*, which is abundant in INU group as per our preceding study^21^. Abundance of ethanol is directly associated with genera *Lactococcus, Bilophila, Akkermansia* and *Faecalibaculum*, while it is inversely associated with genera *Dubosiella* and Lactobacilli group. The higher abundance of ethanol in the PTB group may be partly associated with relatively lower abundance of *Dubosiella* and Lactobacilli group, as observed in our previous report^21^. TMA shows a strong positive association (*p* < 0.01) with *Faecalibaculum, Enterococcus, Akkermansia, Lactococcus, Bilophila,* and *Frisingicoccus,* and a negative association with *Dubosiella*. Choline shows an inverse association with *Enterococcus*, which is linked to sex-specific differences in the PTB group where *Enterococcus* was more prevalent in males than females^21^, and so are the choline levels in this study (Fig. 1C). Among SCFAs, butyrate is positively influenced by *Streptococcus,* Lactobacilli group and *Odoribacter*, while negatively by *Blautia* and *Akkermansia*. Acetate production is positively correlated with *Parasutterella, Barnesiella, Bacteroides,* and *Bilophila*, whereas *Lachnospiraceae_NK4A136* and *Faecalibaculum* decrease acetate abundance. Propionate production is increased with increased abundance of *Barnesiella, Frisingicoccus, Parasutterella, Butyricimonas, Lachnoclostridium, Bilophila,* and *Parabacteroides*.

Further stringent insights into microbiome-metabolome crosstalk using significantly ranked correlation networks (R^2^ = 0.85; *p* < 0.01) demonstrate group-specific alterations in metabolomic profiles as a function of microbiota (Fig. 3B). In the CTL group, an inverse association of amino acids such as leucine, isoleucine, phenylalanine with *CAG-352*, lysine with *Frisingicoccus*, tyrosine with *Holdemania,* and glycine with *Desulfovibrio* is observed. Increased glycine levels in PTB could be associated with decreased *Desulfovibrio*^21^. Besides, *Dubosiella* is positively correlated with propionate and TMA within CTL, while such a relationship is inverse in the LEN group, which may be attributed to changed abundance of microbiota and metabolites in the latter group. In PTB, the balance of acetoin is based on the relative abundance of *Blautia* and *f-Lachnospiraceae;g_uncultured*, while co-occurrence of lactate and valine is associated with *f-Peptostreptococcsceae* and *Phascolarctobacterium*, respectively.

In the BEP group, the abundance of *Phascolarctobacterium* is directly associated with propionate and butyrate, while *Turicibacter* is associated with serine metabolism, and *Adlercreutzia* and *Dubosiella* are associated with xanthine metabolism. Within the LEN group, acetate is positively associated with *Parasutterella*, TMA with *Faecalibaculum*, and valine, formate and phenylalanine with *Adlercreutzia*. Additionally, aspartate and ornithine metabolism in LEN are mutually exclusive with *Odoribacter* and *Lactococcus*, respectively.

The CKP group exhibits a more complex microbiota-metabolite network due to its highest bacterial diversity^21^. The abundance of metabolites, such as acetoin, lactate, and TMA, is directly dependent upon the presence of genera *Enterococcus, Eggerthella, Erysipeatoclostridiaceae*, *Romboutsia,* and *Lachnospiraceae-NK4A136*, many of which are reduced in CKP^21^. Moreover, *Turicibacter* and the latter two genera are negatively associated with glycine metabolism. Furthermore, choline abundance is negatively associated with *Butyrocimonas, Bacteroides,* and *f-Lachnospiraceae;g_uncultured,* the latter two of which are increased for CKP^21^. In the INU group, there is mostly a positive correlation for the microbiota-metabolite network. The production of lactate and acetate is positively influenced by *Intestinimonas* and *f-Lachnospiraceae;g_uncultured.* The latter also impacts TMA along with *Bilophila* and an uncultured family of *o-Rhodospirillales*. Furthermore, the predominance of *Dubosiella* exhibits a direct and inverse influence on fumarate and butyrate production, respectively, which might be the reason behind low butyrate levels in INU group. Also, the accumulation of propionate and glutamate is directly linked to *Barnesiella*, while malonate presence is associated with *Blautia* and *Enterococcus*.

**Figure 4:**
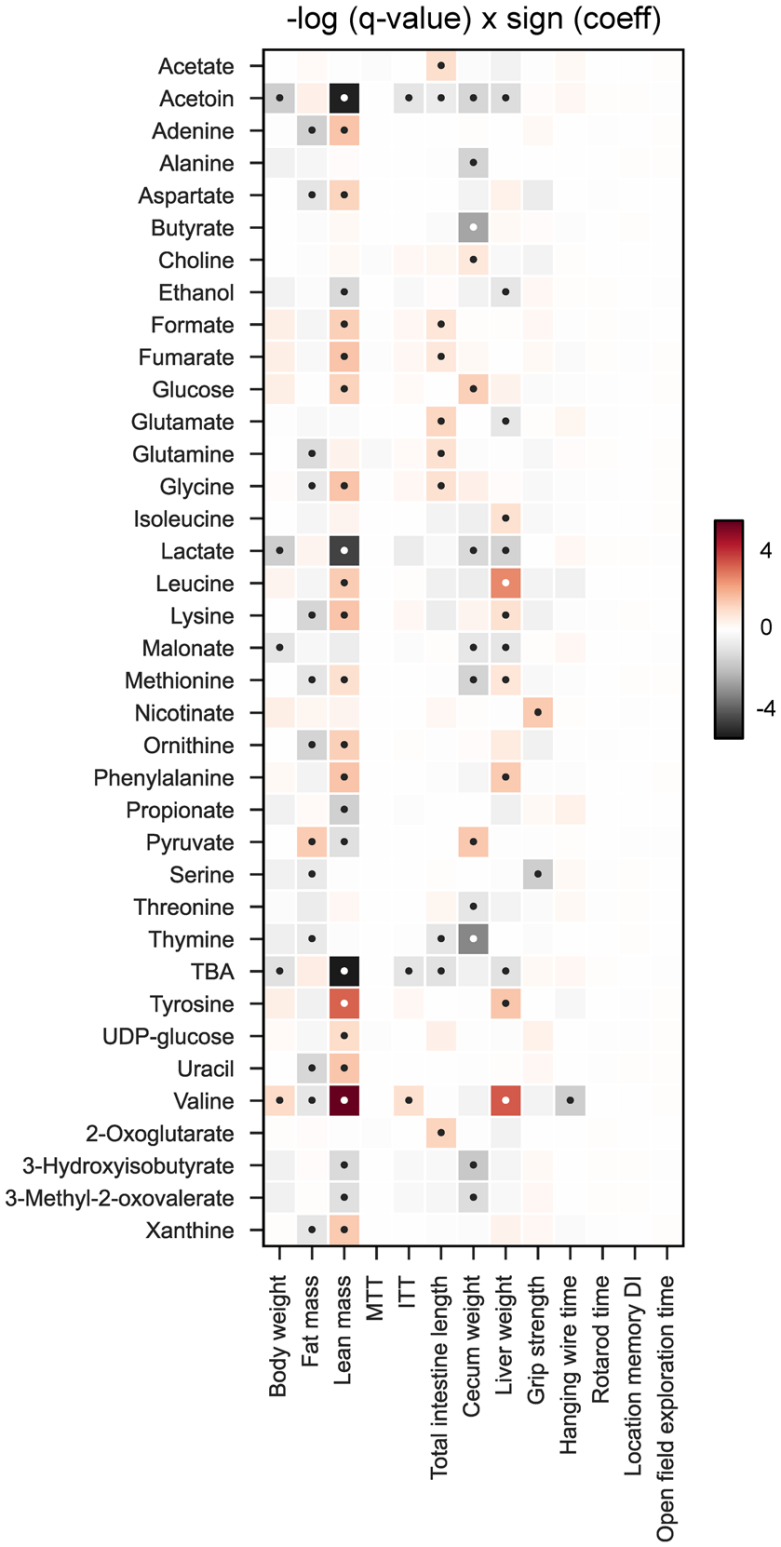
Associations of metabolites with physiological, neurobehavioral, and intestinal tissue parameters. Associations between metabolites and respective parameters were assessed using linear models in MaAsLin 2 (q-value *< 0.25). *MTT* area under curve for meal tolerance test, *ITT* area under curve for insulin tolerance test.

These findings highlight that the intestinal levels of these metabolites are tightly regulated by the complex interplay of metabolic reactions occurring within the gut microbes, which are continuously involved in the biosynthesis of metabolites by one group and its cross-feeding by another group of microbes. Furthermore, we also observe association of several metabolites with previously measured physiological, neurobehavioral, and intestinal tissue parameters^21^ (Fig. 4). Specifically, lean body mass shows the strongest association with metabolites, wherein tyrosine and valine are positively correlated, while lactate, acetoin, and TBAs exhibit an inverse correlation. Additionally, cecum weight positively correlates with choline, glucose, and serine, while thymine and butyrate show an inverse association. Valine and leucine exhibit a positive correlation with liver weight.

### Resistant starches from dietary pulses may impact specific metabolite pathways in the gut

**Figure 5:**
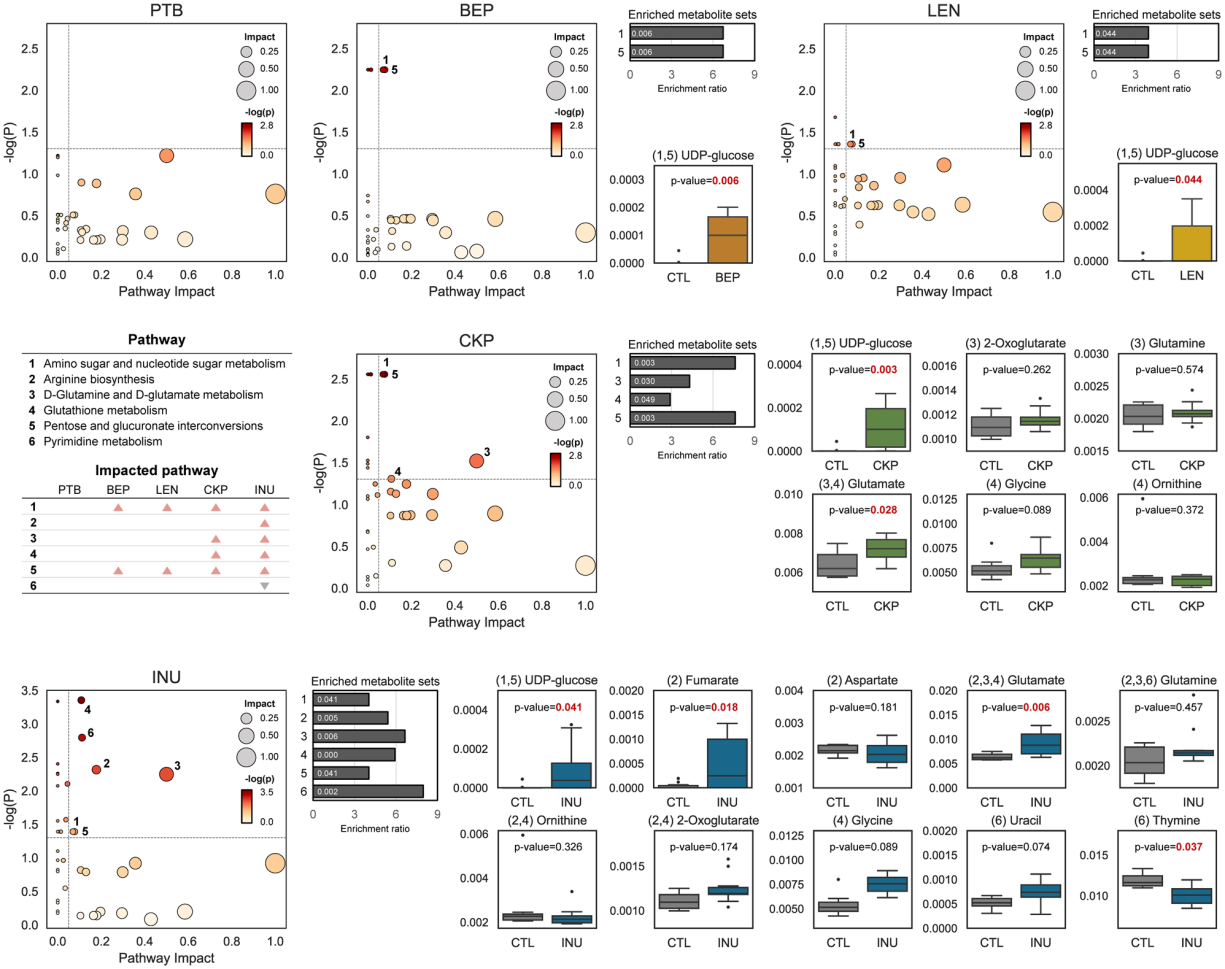
Resistant starches from different dietary pulses may impact specific metabolite pathways in the gut. Metabolic pathway analysis and metabolite set enrichment analysis (MSEA) based on metabolites associated with dietary fiber groups (resistant starch or inulin) relative to standard western-style diet group. Metabolites are mapped to KEGG metabolic pathways. Scatter plots of pathway impact and − log *p* value and relative abundance of metabolites related to significantly enriched metabolic pathway. *CTL* control western-style diet group, *PTB* pinto beans, *BEP* black-eyed peas, *LEN* lentils, *INU* inulin.

Metabolic pathways impacted after RS intervention are summarized in Fig. 5. Our enrichment analyses show that RSs have an impact on six pathways: amino sugar and nucleotide sugar metabolism, arginine biosynthesis, D-glutamine and D-glutamate metabolism, glutathione metabolism, pentose and glucuronate interconversions, and pyrimidine metabolism. All groups except for PTB affect metabolic pathways, with INU having the greatest impact followed by CKP, LEN and BEP. UDP-glucose is the only metabolite significantly enriched in all four RS groups and is involved in amino sugar-nucleotide sugar metabolism and pentose-glucuronate interconversions. In CKP group, glutamate abundance is associated only with the enrichment of D-glutamine and D-glutamate metabolism. In contrast, in INU group, the enrichment of the former pathway, along with arginine biosynthesis, is linked to significant enhancement of glutamate and fumarate metabolites. However, the predictive nature of these metabolic pathways may limit the precise interpretation of the results. Hence, it calls for further comprehensive assessment using more sensitive analytical tools and more inclusive models.

## Discussion

Emerging evidence demonstrates the beneficial effects of dietary fibers on host health by positively modulating the gut microbiome. However, studies that delineate mechanistic insights into microbial metabolic processes occurring in gut milieu during the digestive fermentation of RS are limited. Furthermore, the modulating effects of dietary pulses-derived RS on gut metabolomic pool in ageing milieus remain largely unexplored. Recently, we reported the prebiotic effects of pulses-derived RS on gut microbiome, glucose metabolism, and intestinal function in older mice colonized with human microbiota^21^. Propelled by these compelling findings, we herein aimed to elucidate the shifts in the metabolic function of gut microbiota in these ‘humanized’ mice. As mentioned above, these RS-driven modulations in the metabolomic profiles encompass SCFAs (formate, acetate, butyrate, propionate); hydroxy acids (lactate); aromatic amino acids (phenylalanine, tyrosine), branched-chain amino acids (isoleucine, leucine, valine); carbohydrates (glucose), TCA cycle intermediates (fumarate), nucleosides (UDP-glucose, uracil, xanthine, adenine), ethanol, bile acids, cholesterol, and diet-microbiota originated metabolites (choline-trimethylamine). Some of these metabolites have previously been found to be altered in HFD-induced animal models compared to healthy controls^16,22^. The net abundance of gut metabolites is dictated by the complex ecological events occurring between gut microbes, host epithelial cells, and microbial-host co-metabolisms of indigestible dietary molecules. Metabolites originating from gut microbes dominate the distal gut as metabolites from dietary meals are majorly absorbed in the small intestine^23^. Thus, the distinct RS-specific metabolic outcomes generated by the gut microbiota reported in this study corroborate that even nuanced structural differences in RS may induce divergent gut microbiome-metabolomic signatures^5^.

We observe differential abundance of butyrate upon consumption of LEN and CKP, and of propionate for the INU group. Generally, butyrate production is enhanced in the presence of Firmicutes, while Bacteroidota favor acetate and propionate production^24^. This microbiota-driven metabolite abundance might be explained by the predominant Firmicutes in LEN, while Bacteroidota are dominant in INU^21^. The relatively higher proportion of acetate and propionate in the INU group could be partially explained by the higher abundance of *Parasutterella, Bacteroides,* and *Parabacteroides,* and as well as the lower prevalence of *Lachnospiraceae_NK4A136* and *Faecalibaculum,* as reported in our preceding study^21^. Moreover, propionate biosynthesis at phylum level occurs via two modes: the lactate pathway regulated by Firmicutes and the succinate pathway by Bacteroidota^25^. Our correlational analyses reveal a positive association of propionate with the phylum Bacteroidota and many of its genera, including *Bacteroides* and *Parabacteroides*. Members of these genera are succinate-producers, whereby succinate act as a substrate for other commensals for conversion into propionate^26^, thus suggesting the dominance of the succinate pathway in the INU group. The lower production of butyrate in INU could be due to lower levels of lactate and/or lactate-derived butyrate-producers as lowered lactate-to-butyrate conversion during in-vitro fecal fermentation of fructo-oligosaccharide (FOS) has been reported^27^. Butyrate biosynthesis is regulated by different metabolic pathways, with either acetate or propionate as precursors, and is pH-sensitive, with high production rates observed at low colonic pH values^27^. Although we did not quantify fecal pH levels, it is likely that the relatively higher lactate levels, coupled with Firmicutes abundance in LEN, favored butyrate production. Previous reports have shown a direct association of lactate with butyrate in RS-fed cats^28^. Collectively, variations in fecal SCFAs concentration among different treatment groups could also be ascribed to the cumulative effects of production, absorption, microbial cross-feeding, and complex feedback interactions occurring between bacterial metabolites and host epithelial tissues^29^. Although the beneficial effects of SCFAs on host health have been amply demonstrated in many diseased states, there are instances where abnormally high levels of SCFAs could induce metabolic^30^, immunological^31^ and neurodevelopmental dysregulations^32^. Thus, future research aimed at defining the appropriate (homeostatic) levels and proportions of SCFAs that promote optimal health would help to address this discordance.

Recent studies have elucidated the existence of an intricate relationship between bile acids and the gut microbiome in regulating host metabolism under different pathophysiologies^33,34^. For instance, high levels of primary bile acids have been observed in patients with diarrhea-predominant irritable bowel syndrome^35^. The bile acids-binding capacity of RS could aid in weight management, glycemic index modulation, and cholesterol reduction^36^. In this study, we observe a negative correlation between fecal concentrations of TBAs and cholesterol in all treatment groups compared to the CTL group, with a more pronounced effect exhibited by the INU group. Similarly, Ke et al.^37^ reported an enrichment of TBAs in HFD-induced obesogenic mice, which were later reduced to appreciable levels after a 12-week synbiotic intervention comprising oat β-glucan and probiotic strains of *Bifidobacterium animalis* and *Lactobacillus paracasei*. Besides, the predominance of TBAs in gut favors the growth of gram-negative bacteria over gram-positive ones^38^. This could explain the positive correlation of gram-negative genera (*Butyricimonas, Colidextribacter* and *Odoribacter*) with TBAs and cholesterol in the CTL group. *Colidextribacter* and *Odoribacter* have previously been associated with hypercholesterolemia and epididymal adipose weight, respectively^39,40^ whereas *Butyricimonas* has also been associated with HFD feeding in mice^22^.

The impact of HFD on amino acid metabolism is well documented^16,22^. The CTL group shows enrichment of aromatic amino acids (phenylalanine and tyrosine) and branched-chain amino acids (isoleucine and leucine). Higher abundance of these fecal aromatic amino acids was reported earlier in HFD-fed rats^16^. It is well recognized that gut microbiota degrades these essential amino acids, with certain *Clostridium* species catabolizing phenylalanine to tyrosine and then to 4-hydroxyphenylacetate under anaerobic conditions^41^. The high abundance of glutamine and glutamate in all treatment groups suggests the immunomodulatory potential of RS, as previously reported in our study^21^. Glutamine has been shown to promote IL-10-producing intraepithelial lymphocytes, while glutamate can potentiate immunotolerance in the gut-associated lymphoid tissue^42,43^. The inverse association of methionine with treatment groups may also suggest a beneficial effect, as dietary restriction of methionine has shown to reduce inflammation and improve gut permeability in HFD-fed mice^44^. Interestingly, we also observe a higher abundance of threonine in the LEN group, which suggests a positive impact, as studies have shown that dietary supplementation of threonine could reduce obesity-linked perirenal and epididymal fat^45^. Fecal levels of glycine, a metabolite involved in conjugation of primary bile salts in the liver, were increased in all treatment groups, suggesting its release during the deconjugation of bile salts by gut microbiota. *Bacteroides* are primarily involved in this deconjugation process^46^ and are also found to be associated with glycine in our study (Fig. 3A). However, serum levels of glycine have been reported to increase post HFD-feeding^22^. Nonetheless, dysregulated amino acid metabolism has been previously linked to gut dysbiosis, with serum glycine deficiency implicated in non-alcoholic fatty liver disease^47^. Further investigations are needed to determine whether these changes in the gut are also reflected in the serum metabolome.

TMA, a gut microbiota-derived metabolite, is implicated in exacerbating the risk of cardiovascular diseases. Gut bacteria harboring specific enzyme complexes (e.g., CutC/D and CntA/B) have the ability to liberate TMA from high-fat foods containing TMA moieties such as choline, phosphatidylcholine, and L-carnitine, which is converted into the proatherogenic trimethyl amine N-oxide (TMAO) by hepatic flavin-containing monooxygenase (FMO) enzymes^48,49^. Choline is positively correlated with all the treatment groups except PTB, which is expected because the basal western-style diet itself contains small amounts of choline and fat sources (e.g., lard) (see supplementary Table S1 online). Apart from its involvement in TMA metabolism, choline is considered essential for the host as it serves as a precursor for neurotransmitter acetylcholine and facilitates the biosynthesis of cellular phospholipid membrane^50^. The negative association of choline with PTB might be related to high prevalence of *Enterococcus* in this group, which in turn showed a strong inverse association with choline. Some *Enterococcus* taxa have been reported to carry the choline TMA-lyase gene (cutC) ^51^. Interestingly, this genus also showed positive correlation with TMA production, pointing towards choline-to-TMA conversion in PTB. Furthermore, the results showed that TMA had a positive correlation with INU, while it was only weakly or inversely associated with LEN and CKP. These findings suggest that the latter two RSs may play a role in suppressing the choline-to-TMA metabolism by restructuring the gut microbiome. The role of TMA-derived TMAO in cardiovascular outcomes is still debatable as it could also have beneficial impact on the host by promoting protein stabilization through activating its compensatory stress response action^52^. Nonetheless, it should be an interesting topic for further studies to examine the plasma TMAO levels and cardiovascular health markers among such interventions to clarify its plausible harmful and protective mechanisms.

In addition, we identify varying concentrations of several intermediate metabolites such as lactate, acetoin, pyruvate, ethanol, UDP-glucose, and others. The net production of these metabolites depends on the complex interplay between different gut microbiota species through fermentative glycolytic pathways and nucleotide sugar metabolisms. Of these metabolites, UDP-glucose was significantly enhanced in the BEP and CKP groups. Although the exact role of UDP-glucose in RS intake is unclear, it has been previously implicated in modulating gastric motility^53^ and improving hepatic insulin sensitivity by facilitating the incorporation galactose into glycogen synthesis^54^. Ethanol is another endogenous metabolite produced during the heterofermentative cycle of many gut microbes, which can reach the liver and get converted into acetate and acetaldehyde^55^. We observe a positive association of ethanol in the PTB group, presumably due to the lower abundance of the *Dubosiella* and Lactobacilli group, which have previously been found to be reduced in alcoholic liver injury models but restored after treatment with Antrodin A, extracted from the mycelium of *Antrodia camphorate* fungus^56^.

In our previous study, we reported that the treatment groups (especially LEN, CKP, and INU) increased the abundance of *Dubosiella*, while concomitantly reducing *Faecalibaculum*^21^. This trend has also been reported in a preclinical study involving resistant dextrin supplementation in HFD^57^. Interestingly, the metabolites (acetoin, lactate, trimethylamine, and ethanol) which showed a negative associated with *Dubosiella* exhibited a positive association with *Faecalibaculum*. On the other hand, the correlation of other metabolites (adenine, glycine, uracil, and valine) was positively linked with *Dubosiella* but negatively with *Faecalibaculum*. Little is known about the association of these taxa with gut metabolites, as both taxa belonging to *Erysipelotrichaceae* were recently discovered^58^. Nonetheless, recent studies have shown a positive association of *Faecalibaculum* with markers of hepatic insult, such as malondialdehyde, triacylglycerols, and alanine/asparate aminotransferases^56^. Additionally, our findings partially align with previous studies^59^ that reported a direct association of TMA production with both *Dubosiella* and *Faecalibaculum*. We also observe a positive association of *Bilophila*, a potential pathobiont, with TMA and ethanol, which aligns well with earlier studies^60,61^. Furthermore, we find a positive correlation of *Akkermansia* with TMA, ethanol, and acetoin while butyrate is negatively correlated. The association of plasma TMA with *A. muciniphila* has been recently reported in diet-induced obesity models^62^. Although the beneficial role of *A. muciniphila* in ameliorating obesity-associated metabolic dysfunction, improving glucose and lipid metabolism, along with intestinal immunity, has been documented^63–65^, studies demonstrating its negative association with specific aspects of the host health are also available. Recently, the adverse effects of supplementing *A. muciniphila* post-antibiotic treatment in mice has been shown to exacerbate colonic tumor burden^66^. Moreover, fecal abundance of *A. muciniphila* in a chronic stress-induced mouse model of Parkinson’s disease has been found to be increased along with decreased fecal butyrate and increased serum lipopolysaccharide levels^67^. The inverse association of *Akkermansia* with butyrate could be explained by its mucin metabolism into propionate and acetate and its lack of genes involved in butyrate production^25,66^. However, it might indirectly promote butyrate production by supporting the growth of non-mucin butyrate-producing taxa from families *Ruminococcaceae* and *Lachnospiraceae*^25^, which may have enhanced butyrate production in LEN and CKP groups wherein the members of these two families were increased^21^. These contrasting effects of *A. muciniphila* on the intestinal health of the host can be attributed to the strain-level phylogenetic differences, which are closely linked to its distinct functional and metabolic features^68^.

## Conclusions

To our knowledge, this study is the first to report on the specific modulations induced by resistant starches from various dietary pulses in the gut metabolome and microbiome-metabolome interactions within ageing gut milieus. The phenotypic differences observed in the gut microbiome-derived metabolites are closely correlated with the production of SCFAs and the altered metabolism of bile acids and amino acids. More specifically, the levels of butyrate are correlated with the intake of LEN and CKP, while propionate production is correlated with INU intake. Through integrated multi-omics correlational analyses of microbiome-metabolome arrays, we reveal complex RS-specific mutualistic and competitive interactions occurring across different taxa and metabolites. This highlights the potential of discrete structures of dietary pulses-based fibers in inducing targeted modulation of the gut metabolomic pool. Our study provides novel and valuable information on the mechanistic understanding of NMR-based metabolomic function of the gut microbiome in mitigating obesity-related disorders. Further studies utilizing other comprehensive metabolomics approaches (e.g., LC–MS, GC–MS), as well as metatranscriptomics and metaproteomics approaches, are necessary to validate and provide deeper insights into gut microbial metabolites in host-metabolic pathways, thereby ascertaining their precise functional consequences.

## Materials and methods

### Extraction and preparation of RS from pulses

Starch extraction from pulse seeds was performed in accordance with our previously described method^69^. RS was obtained via simulated gastric digestion from purified starch as previously described by Tuncil et al.^70^ with slight modifications. Briefly, 12 g of starch were gelatinized in 240 mL sodium phosphate buffer (pH 6.9) and cooled to 37 °C, followed by incubation for 15 min in presence of two mL of salivary amylase (Sigma-Aldrich). Hydrolysis was carried out under continuous stirring, and the pH was adjusted from 6.9 to 2.0 using 6 M HCl. Subsequently, digestion was initiated using sequential steps of enzymes addition: pepsin (37 °C, pH 2.0, 30 min) and four mL pancreatin (37 °C, pH 6.9, 90 min). The hydrolyzed starch was dialyzed (6–8 kDa, 36 h) and the leftover undigested starch was freeze dried for 72 h.

### Animal studies

Animal experimentation was conducted as per our previous protocol^21^. Briefly, the native gut microbiota of 55 weeks old C57BL/6J mice was depleted and cleansed via four days of ad-libitum feeding with an antibiotic cocktail (ampicillin [1 g], Metronidazole [1 g], Neomycin [1 g], and Vancomycin [0.5 g] per liter of drinking water), followed by a four-hour fast before administration of four doses of oral gavage with polyethylene glycol (200 μL per dose; 425 g/L). Thereafter, fecal samples from five human donors (age: 50–55 years) were pooled and transplanted into mice as described previously^21^. Mice were randomly allocated into six groups (n = 14–16/ group; 7–8 for each sex) based on a 20-week dietary intervention: CTL (western-style high-fat diet control), four treatment groups containing CTL diet supplemented with RS (5% w/w) from pinto-beans (PTB), black-eyed peas (BEP), lentils (LEN), and chickpeas (CKP), and one positive control (INU) containing 5% w/w inclusion of inulin in the CTL diet, in line with our previous studies^71^. Fecal samples for metabolome and microbiome analysis were collected and stored at − 80 °C until further analysis. Mouse experiments are described according to ARRIVE guidelines (https://arriveguidelines.org).

### Metabolomics analysis

Fecal samples from the control and treatment groups were extracted using water according to a previously described protocol^72^ with minor changes. Briefly, samples were extracted by vertexing for 5 min with deionized water. The extracted samples were then mixed with a phosphate buffer (pH = 7.4) in D_2_O to make a final solution containing 10% D2O, 0.1 M phosphate, and 0.1 mM Trimethylsilyl propionate (TSP). After centrifugation, the samples were transferred to a 5 mm NMR tubes for data acquisition using a Bruker Ascend 400 MHz high-resolution NMR (Bruker Biospin, Germany. A 1D first increment of a NOESY (noesygppr1d) experiment with water suppression was applied to all samples with 64 scans. All NMR spectra were phased and referenced to TSP in TopSpin 4.06 (Bruker BioSpin, Germany). NMR processing was carried out in Amix 4.0 (Bruker BioSpin) and the NMR spectra were bucketed using our previously reported automatic method^73^ to minimize peak overlap and splitting. Metabolite indentation was carried out using Chenomx 8.6 (Chenomx Inc). Total intensity normalization was applied before further data analysis. The raw dataset containing quantitative information of identified metabolites for each sample in this study can be retrieved from the supplementary material.

### Gut microbiome analysis

The gut microbiome was measured according to our previously described methods^3,71,74–78^. Genomic DNA was extracted from 200 mg of the fecal specimen using the QIAmp PowerFecal Pro DNA Kit (Qiagen) following the manufacturer's instructions. The hypervariable V4 region of the bacterial 16S rRNA gene were amplified using Universal primers 515F (barcoded) and 806R in accordance with the Earth Microbiome Project benchmark protocol (https://earthmicrobiome.org/). The library was pooled at equal molar concentrations and sequenced for paired-end (2 × 300 bp) sequencing using an Illumina MiSeq sequencer (using Miseq reagent kit v3; Illumina Inc., San Diego, USA). Microbiome bioinformatics analysis was conducted using QIIME2 (ver. 2-2022.8) ^79^. Raw sequence demultiplexing, filtering, trimming and denoising qwew carried out through DADA2^80^. All identified amplicon sequence variants (ASVs) were aligned with the MAFFT^81^ andASVs were assigned with a naïve Bayes taxonomy classifier developed for the sklearn classifier against the pre-built from the 99% SILVA 138 database^82,83^.

### Bioinformatics and statistical analysis

Metabolome analyses were executed using ‘R’ or ‘Python’ packages. To explore and visualize differences between the CTL and RS-treated groups, a PCoA based on Bray–Curtis dissimilarity was conducted, and statistical significance was assessed using the PERMANOVA^84^ with 999 random permutations. To identify the most predictive metabolites, supervised classification was performed with the q2-sample-classifier plugin for QIIME2 via nested stratified fivefold cross-validation with Random Forest^85^ classifier grown with 1,000 trees. STAMP v 2.1.3 software^86^ was explored to compare the difference in mean proportion of 95% confidence intervals between the CTL and RS-treated groups. Linear discriminant analysis (LDA) effect size (LEfSe)^87^ was used to identify the difference in metabolites, and Human Metabolome Database (HMDB) chemical taxonomy^88^ is utilized to assign metabolites and depict taxonomic cladogram. A network between the bacterial taxa and metabolites was constructed by calculating the Spearman correlation and significant associations (Spearman correlation coefficient > 0.85 and Benjamini–Hochberg corrected *p* value < 0.01) were visualized using Cytoscape v3.9.1^89^. The association between metabolites and physiological, neurobehavior, and intestinal tissue measures were analyzed using multivariate association analysis, MaAsLin2^90^. The benjamini–hochberg corrected p-value (q-value) threshold was set to 0.25. Metabolic analysis and MSEA based on the Kyoto Encyclopedia of Genes and Genomes (KEGG) *Mus musculus* library were performed with MetaboAnalyst v5.0^91^. The enrichment method and topology analysis are conducted using the global test and relative-betweenness centrality in metabolic analysis.

### Ethics approval

This study was carried out in accordance with the guidelines of the Institutional Animal Care and Use Committee. The protocol was approved by the Institutional Animal Care and Use Committee at Florida State University (PROTO202100008).

## Supplementary Information

Supplementary Information 1.

Supplementary Information 2.

## Data Availability

All datasets generated for this study are included in the manuscript/supplementary files. All the raw sequencing datasets are deposited in the NCBI Sequence Read Archive (SRA) public repository database under SRA BioProject number PRJNA902407.
