# Supplementary material for: Resistant starches from dietary pulses modulate the gut metabolome in association with microbiome in a humanized murine model of ageing
Source: Sci Rep. 2023 Jun 29;13:10566. doi: 10.1038/s41598-023-37036-w (PMC10310774; doi:10.1038/s41598-023-37036-w)
Supplement: Supplementary file 1 — Supplementary Information 1. [file 41598_2023_37036_MOESM1_ESM.docx]

**Resistant starches from dietary pulses modulate the gut metabolome in association with microbiome in a humanized murine model of ageing**

Saurabh Kadyan^1^, Gwoncheol Park^1^, Bo Wang^2^, Prashant Singh^1^, Bahram Arjmandi^1^, Ravinder Nagpal^1^*

^1^Department of Nutrition and Integrative Physiology, College of Health and Human Sciences, Florida State University, Tallahassee, Florida 32306, USA.

^2^Department of Biomedical and Chemical Engineering and Sciences, Florida Institute of Technology, Melbourne, Florida 32901, USA.

*Correspondence: [rnagpal@fsu.edu](mailto:rnagpal@fsu.edu)

**Supplemental Table S1: Diet composition used in present study.**

|  | CTL | PTB | BEP | LEN | CKP | INU |
| --- | --- | --- | --- | --- | --- | --- |
| Ingredient, gm |  |  |  |  |  |  |
| Casein | 38.5 | 38.5 | 38.5 | 38.5 | 38.5 | 38.5 |
| Fish Protein Isolate | 8.5 | 8.5 | 8.5 | 8.5 | 8.5 | 8.5 |
| Egg white | 55 | 55 | 55 | 55 | 55 | 55 |
| Beef, cooked, powdered | 77 | 77 | 77 | 77 | 77 | 77 |
| L-cystein | 3 | 3 | 3 | 3 | 3 | 3 |
| Corn starch | 30 | 25 | 25 | 25 | 25 | 28 |
| Wheat starch | 195 | 168 | 168 | 168 | 168 | 180 |
| Potato Starch | 30 | 25 | 25 | 25 | 25 | 28 |
| RS-PTB | 0 | 44 | 0 | 0 | 0 | 0 |
| RS-BEP | 0 | 0 | 44 | 0 | 0 | 0 |
| RS-LEN | 0 | 0 | 0 | 44 | 0 | 0 |
| RS-CKP | 0 | 0 | 0 | 0 | 44 | 0 |
| Inulin | 5 | 5 | 5 | 5 | 5 | 45 |
| Sucrose | 205 | 205 | 205 | 205 | 205 | 205 |
| Fructose | 22 | 22 | 22 | 22 | 22 | 22 |
| Cellulose | 18 | 18 | 18 | 18 | 18 | 18 |
| Menhaden oil | 1 | 1 | 1 | 1 | 1 | 1 |
| Butter, anhydrous | 54 | 54 | 54 | 54 | 54 | 54 |
| Lard | 34 | 34 | 34 | 34 | 34 | 34 |
| Flaxseed oil | 1 | 1 | 1 | 1 | 1 | 1 |
| Olive oil | 28 | 28 | 28 | 28 | 28 | 28 |
| t-BHQ | 0.005 | 0.005 | 0.005 | 0.005 | 0.005 | 0.005 |
| Mineral mix | 10 | 10 | 10 | 10 | 10 | 10 |
| Dicalcium phosphate | 13 | 13 | 13 | 13 | 13 | 13 |
| Calcium carbonate | 5 | 5 | 5 | 5 | 5 | 5 |
| Potassium Citrate | 16 | 16 | 16 | 16 | 16 | 16 |
| Vitamin mix | 10 | 10 | 10 | 10 | 10 | 10 |
| Biotin | 0.1 | 0.1 | 0.1 | 0.1 | 0.1 | 0.1 |
| Choline | 2 | 2 | 2 | 2 | 2 | 2 |
| Cholesterol | 1.5 | 1.5 | 1.5 | 1.5 | 1.5 | 1.5 |
| Kcal |  |  |  |  |  |  |
| Protein | 547 | 547 | 547 | 547 | 547 | 547 |
| Carbohydrate | 1824 | 1824 | 1824 | 1824 | 1824 | 1824 |
| Fat | 1289 | 1289 | 1289 | 1289 | 1289 | 1289 |
| Total | 3661 | 3661 | 3661 | 3661 | 3661 | 3661 |
| Kcal% |  |  |  |  |  |  |
| Protein | 15 | 15 | 15 | 15 | 15 | 15 |
| Carbohydrate | 50 | 50 | 50 | 50 | 50 | 50 |
| Fat | 35 | 35 | 35 | 35 | 35 | 35 |
| % Added RS | 0 | 5 | 5 | 5 | 5 | 0 |
| % Inulin | 0.5 | 0.5 | 0.5 | 0.5 | 0.5 | 5 |
| Kcal/g | 4.2 | 4.2 | 4.2 | 4.2 | 4.2 | 4.2 |
